# A single-center experience of over 300 cases of single-incision robotic cholecystectomy comparing the da Vinci SP with the Si/Xi systems

**DOI:** 10.1038/s41598-023-36055-x

**Published:** 2023-06-10

**Authors:** Yoo Jin Choi, Nguyen Thanh Sang, Hye-Sung Jo, Dong-Sik Kim, Young-Dong Yu

**Affiliations:** 1 Division of HBP Surgery and Liver Transplantation, Department of Surgery, Korea University College of Medicine, 73 Goryeodae-ro Seongbuk-gu, Seoul, 02841 Korea; 2 Department of Surgery, Trung Vuong Hospital, Ho Chi Minh City, Vietnam

**Keywords:** Gall bladder, Preclinical research, Diseases, Medical research, Oncology

## Abstract

Minimally invasive surgery is usually more beneficial than open surgeries in various fields of surgery. With the newly developed Single-Port (SP) robotic surgical system, even single-site surgery has become easier to access. We compared single-incision robotic cholecystectomy between the Si/Xi and SP systems. This retrospective single-center study enrolled patients who underwent single-incision robotic cholecystectomy between July 2014 and July 2021. The clinical outcomes of the da Vinci Si/Xi and SP systems were compared. In total, 334 patients underwent single-incision robotic cholecystectomy (118 Si/Xi vs. 216 SP). The SP group had more chronic or acute cholecystitis than the Si/Xi group did. There was more bile spillage in the Si/Xi group during the surgery. The total operative and docking times were significantly shorter in the SP group. There was no difference in the postoperative outcomes. The SP system is safe and feasible regarding comparable postoperative complication rates and is more convenient regarding docking and techniques.

## Introduction

The robotic surgical system, which will be a leading surgical technique in the future, was first developed and commercialized in 1997. This robotic system has been gradually developed by improving the limitations of the previous system, from the first da Vinci model to the latest da Vinci SP. Single-incision surgery in the robotic surgical field became possible with the introduction of the da Vinci Si model in 2009. Additionally, it was in the spotlight at that time because there were several restrictions on single-incision laparoscopic surgery.

The da Vinci Si and Xi in single incision surgeries reduced internal collision of the instruments and external collision of surgeons’ hands by using the triangulation of instruments^1^ and eliminated potential collision between the operator and the camera assistant by allowing control of the camera by the operator himself. However, there was still an element of discomfort using the Si and Xi systems. Contrary to the da Vinci Si and Xi multiport instruments, one of the biggest limitations was the lack of endo-wrist movement in the instruments. Similar to the disadvantages seen in single-incision laparoscopic surgery, they are not fully ergonomic. In addition, these systems require assistance for the lateral traction of the gallbladder.

In 2018, the fourth-generation da Vinci surgical system, the da Vinci SP system, was launched. It was developed for advanced single-incision and narrow-space surgeries. The system includes three robotic arms, each with multiple joints, wrist, and elbow, and the first fully wristed three-dimensional high-definition camera. Applying this system to cholecystectomy for the first time, Cruz et al. demonstrated that robotic SP cholecystectomy was feasible, safe, and effective, and showed better perioperative outcomes than robotic Si cholecystectomy. Moreover, da Vinci SP has been used in more complex hepatobiliary and pancreatic surgeries, such as distal pancreatectomy^2^.

To date, only a few studies have compared the perioperative and postoperative outcomes of different robotic systems during robotic cholecystectomy. Recently, two reports have compared robotic cholecystectomy. One study compared the SP to the Si system in 30 consecutive patients^3^, and another multicenter study compared SP to the Xi system^4^. However, the study cohort that underwent cholecystectomy in these studies was relatively small. To date, this study is the largest series comparing Si/Xi versus SP with three times as many SP cases as in previous studies.

This study aimed to investigate the clinical advantages of the SP system in a large patient cohort by comparing the clinical outcomes between the new SP system and previous Si/Xi systems.

## Methods

### Patients

A total of 334 patients underwent single-incision robotic cholecystectomy between July 2014 and July 2021 at a single tertiary referral center. The data were prospectively collected and retrospectively analyzed. The follow-up periods were 5.37 ± 2.57 months with range from 0 to 35 months.

As robotic surgery is more expensive than open or laparoscopic surgery, not all patients with gallbladder disease undergo robotic cholecystectomy. In general, robotic cholecystectomy is recommended for patients with no or minimal inflammation of the gallbladder. All patients who underwent robotic cholecystectomy using either the Si/Xi or SP system were included in this study. Patients who previously underwent upper abdominal surgery were not recommended for single-incision robotic surgery because of the possible conversion to multiport laparoscopic or open cholecystectomy owing to postoperative adhesions.

Before the SP system was introduced at our center in April 2020, all robotic cholecystectomies were performed using either the Xi or Si system. Subsequently, all subjects underwent single-incision robotic cholecystectomy only with the SP system because the surgeons felt more comfortable with the SP system. This retrospective study was approved by the institutional review board of Korea University Anam Hospital (#2022AN0151). The waived informed consent was approved by IRB of Korea University Anam Hospital. All methods were performed in accordance with the relevant guidelines and regulations.

### Time measurement

The docking, console, and total operative time were measured during the operation. The docking time represented the time interval from the incision to the end of docking of the robotic arm to the cannula. The console time was defined as the time spent by the surgeon at the console during the robotic procedure. The total operative time was defined as the time interval from incision to wound closure.

### Procedure

Procedures with both the Si/Xi and SP systems shared similar surgical procedures, except for the docking techniques. Under general anesthesia, the patient was placed in the supine position. A trans-umbilical incision of approximately 3 cm was made and different ports were placed in the wound.

**Figure 1:**
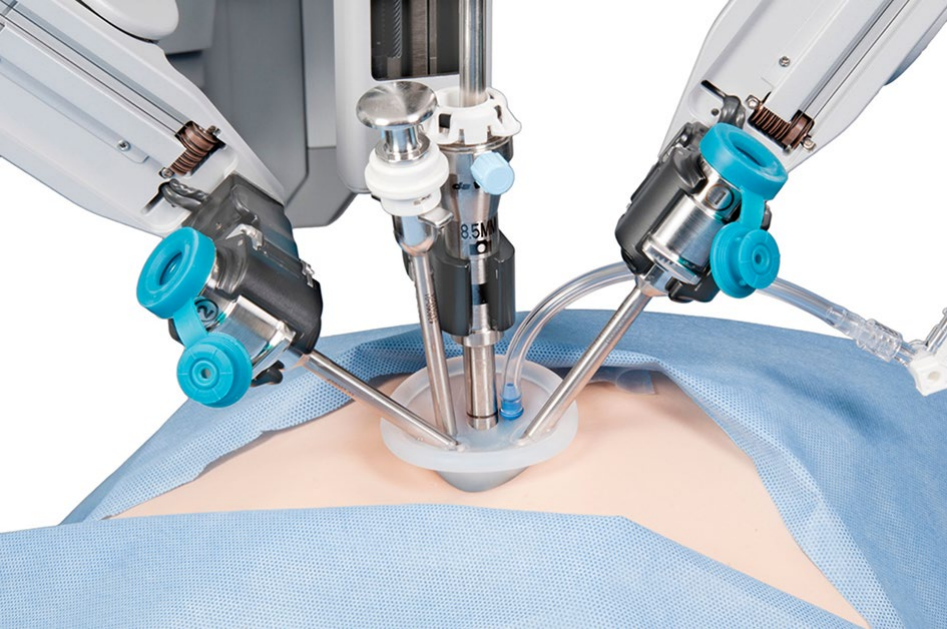
Robotic ports insertion diagram in the single incision surgery with Si or Xi system. The middle port is for the camera. It can be observed that the two side ports have crossed each other. Thus, the right port is for the grasper and the left port is for the hook or hemo-lock clips.

In the Si/Xi system, a Single-site Port ® (Intuitive Surgical, Sunnyvale, California, USA) was placed in the umbilical incision. After a pneumoperitoneum was achieved with 12–15 mmHg, the patients were positioned in reverse Trendelenburg with the right side up, one straight robotic cannula for the camera was inserted in the middle, and two curved-designed robotic cannulas were inserted on the left and right sides. It was ensured that these two curved cannulas crossed each other with the right cannula placed on the left cannula. Subsequently, the assistant inserted the gallbladder grasper through the port and grabbed the GB for retraction. The robot was docked to the cannulas, and the robotic camera was inserted, followed by the insertion of instruments with bending flexibility. The hook was inserted into the left cannula and a grasper was inserted to the right (Fig. 1). The robotic software reconfigures the right and left sides, such that surgeons can move normally using hooks with the right hand and graspers with the left hand. These two curved cannulas allow for internal triangulation, which maximizes the range of motion.

**Figure 2:**
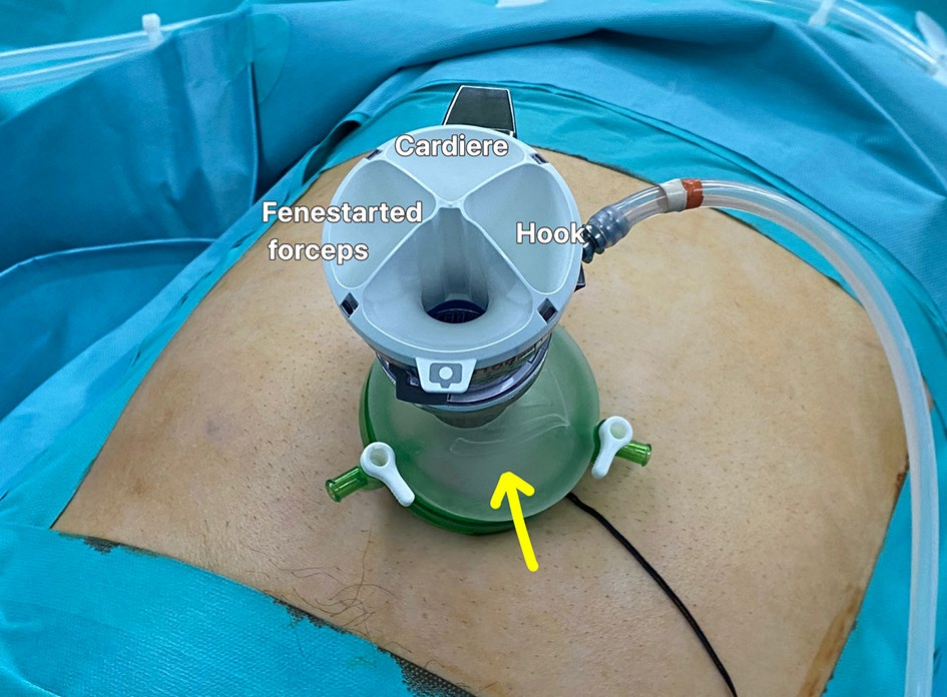
Robotic arms position in the SP robotic system.

**Figure 3:**
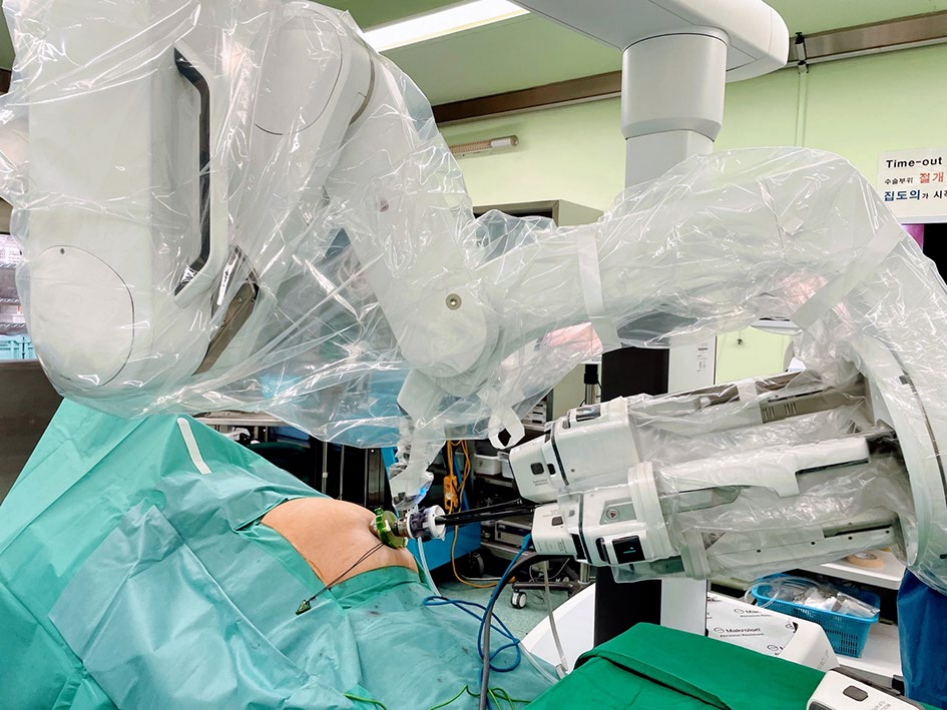
The patient was positioned in reverse Trendelenburg and right-up position. The robot was docked to the cannula with the insertion of the robotic camera and three robotic arms.

In the SP system, the gel port® was placed in the umbilical incision, and pneumoperitoneum and patient positioning were performed in the same manner as with Si/Xi. A 25-mm SP cannula was inserted through the gel port, followed by the insertion of the SP multi-channel guide port into the cannula (Fig. 2). Subsequently, the robot, placed on the left side of the patient, was docked to the cannula, and the robotic camera and three robotic arms were inserted into the cannula (Fig. 3). In the multi-channel guide port, the camera was placed at the lower side, a hook at the right arm, fenestrated bipolar forceps at the left arm, and Cadiere forceps at the upper side. Since the SP system itself had the third arm, cardiac forceps, for GB retraction, there was no need for an assistant.

Afterward, the cholecystectomy and wound closure procedures for both systems were similar, including dissection around Calot’s triangle, ligation of the cystic duct and artery with robotic hemolocks, and dissection of the GB from the liver bed.

### Intraoperative findings

There were four types of intraoperative findings on GB. Adhesion in Calot’s triangle referred to omental adhesions around GB. Acute inflammation was noted if GB was distended or edematous with or without gangrenous change of the wall. This finding was different from the preoperative radiological diagnosis of acute cholecystitis in Table 1. Wall thickening was observed when GB was cut open after the operation. Bile spillage referred to the tearing of GB during the dissection of GB from the liver.

**Table 1:** Demographics.

Variables	Si/Xi (n = 118)	SP (n = 216)	Total (N = 334)	P-value
Age (year)	44.14 ± 11.41	47.34 ± 11.26	46.21 ± 11.40	0.014
Sex, male (%)	32 (27.1%)	94 (43.5%)	126 (37.7%)	0.003
BMI (kg/m^2^)	23.85 ± 3.95	27.09 ± 27.61	25.95 ± 22.36	0.205
ASA (%)				0.286
0	1 (0.8%)	0	1 (0.3%)	
1	98 (83.1%)	188 (87.0%)	286 (85.6%)	
2	19 (16.1%)	28 (13.0%)	47 (14.1%)	
Previous lower abdominal surgery	6 (5.1%)	4 (1.9%)	10 (3.0%)	0.195
Preoperative diagnosis				0.014
Polyp	56 (47.5%)	63 (29.2%)	119 (35.6%)	
Gallstone	59 (50.0%)	139 (64.4%)	198 (59.3%)	
Polyp and stone	2 (1.7%)	7 (3.2%)	9 (2.7%)	
Chronic cholecystitis	0	2 (2.3%)	2 (0.6%)	
Acute cholecystitis	1 (0.8%)	5 (2.3%)	6 (1.8%)	
Preoperative ERCP	2 (1.7%)	7 (3.2%)	9 (2.7%)	0.404

BMI, body mass index; ASA, American Society of Anesthesiology; ERCP, endoscopic retrograde cholangiopancreatography.

### Statistical analysis

Continuous variables are presented as mean ± standard deviation. Categorical variables are expressed as proportions and analyzed using the chi-squared or Fisher's exact test. All statistical analyses were performed using SPSS version 26.0 (IBM Corp., Armonk, NY, USA).

## Results

The Si/Xi and SP groups included 118 and 216 patients, respectively (Table 1). There were significantly more women in the SP group; however, there were no significant differences in age, body mass index (BMI), American Society of Anesthesiology (ASA) score, previous lower abdominal surgery, or preoperative ERCP performance between the Si/Xi and SP groups. The total mean age was 46.21 ± 11.40 years and the mean BMI was 25.95 ± 22.36 kg/m^2^. All the patients were ASA I or II. None of the patients underwent previous upper abdominal surgery, except for one patient who had open abdominal surgery for duodenal perforation, and only 3% had previous lower abdominal surgery. A preoperative diagnosis showed significant differences between the two groups, with the Si/Xi group having more patients with simple polyps and the SP group having more patients with multiple gallstones, and chronic or acute cholecystitis cases. Moreover, additional patients in the SP group underwent preoperative ERCP for CBD stones.

### Operative outcomes

**Figure 4:**
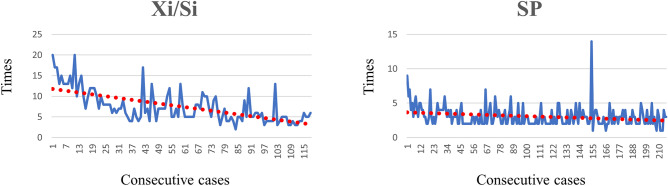
Docking times in chronological order.

All defined operative times demonstrated significant statistical differences. SP robotic cholecystectomy took less docking and console (including the dissection time) times (Table 2). The docking time of the Si/Xi system was 20 min in the first case and was decreased with more consecutive cases, whereas that of the SP system maintained constant inclination (Fig. 4).

**Table 2:** Intraoperative findings and operative times.

Variables	Si/Xi (n = 118)	SP (n = 216)	Total (N = 334)	P-value
Adhesion in Calot’s triangle	9 (7.6%)	12 (5.6%)	21 (6.3%)	0.456
Acute inflammation	3 (2.5%)	7 (3.2%)	10 (3.0%)	0.720
Wall thickening	15 (12.7%)	34 (15.7%)	49 (14.7%)	0.455
Bile spillage	15 (12.7%)	9 (4.2%)	24 (7.2%)	0.004
*Conversion rate	1 (0.8%)	2 (0.9%)	3 (0.9%)	0.714
Docking time (min)	7.50 ± 3.87	3.07 ± 1.46	4.63 ± 3.34	< 0.000
Console time (min)	29.58 ± 14.92	21.18 ± 12.91	24.15 ± 14.21	< 0.000
Actual dissection time (min)	37.08 ± 17.49	24.25 ± 13.46	28.78 ± 16.20	< 0.000
Total operative time (min)	61.06 ± 21.89	21.89 ± 16.10	51.41 ± 19.66	< 0.000

* Conversion to laparoscopic or open.

* EBL: all were less than 50 ml.

Three patients underwent conversion surgery from robotic to laparoscopic and open surgery: one in the Si/Xi group and two in the SP group. Two cases were converted to multiport laparoscopic surgeries because one had acute cholecystitis and the other had severe adhesions from previous upper abdominal surgery. The other case was converted to open surgery because intraoperative findings could not rule out GB cancer. There were no significant differences in the adhesion in Calot’s triangle and wall thickening, which commonly indicate the presence of inflammation. The actual intraoperative findings of acute inflammation did not differ significantly. However, the Si/Xi group had more bile spillages than the SP group (12.7% Si/Xi vs. 4.2% SP., P = 0.004). The estimated blood loss for all patients was < 50 ml, except for cases that were converted to laparoscopic or open surgery. There were no intraoperative complications, such as massive bleeding or bile duct injuries.

### Postoperative outcomes

Postoperative outcomes, including postoperative complications and length of hospital stay, did not show significant differences (Table 3). The rate of postoperative complications in all cases was 11.1%, with 11.9% in the Si/Xi group and 10.6% in the SP group. The most common complications were wound problems, including seroma and infection (8.5% Si/Xi vs. 8.3% SP). The incisional hernia rate was only 1.5%, with 1.7% in the Si/Xi and 1.4% in the SP. One case of fluid collection in the abdominal cavity required percutaneous drainage in the SP group.

**Table 3:** Postoperative outcomes.

Variables	Si/Xi (n = 118)	SP (n = 216)	Total (N = 334)	P-value
Postoperative complication	14 (11.9%)	23 (10.6%)	37 (11.1%)	0.910
Wound seroma	4 (3.4%)	7 (3.2%)	11 (3.3%)	
Wound infection	6 (5.1%)	11 (5.1%)	17 (5.1%)	
Incisional hernia	2 (1.7%)	3 (1.4%)	5 (1.5%)	
Operative site fluid collection	0	1 (0.5%)	1 (0.3%)	
Postoperative stay (day)	2.43 ± 0.947	2.39 ± 0.71	2.41 ± 0.80	0.674

## Discussion

Minimally invasive surgery is entering the era of robotic surgery, beyond the era of laparoscopic surgery. Robotic surgery has overcome the non-ergonomic limitations of laparoscopic surgery and makes MIS possible in several complicated surgeries. Robotic systems have also been developed gradually, and even SP robot systems that can operate with a single hole with minimal invasiveness have been developed. Since the da Vinci SP was launched in 2018, it has only been applied in a few surgical fields. Single-incision surgeries themselves have not received attention because of the risk of surgeries and challenging techniques and have not been popularized to develop more. However, there are several advantages of the SP system in overcoming the potential limitations of single-incision surgeries. We anticipate that our experience with SP robotic cholecystectomy and some previous studies will enable us to pursue MIS with easier techniques and patient safety.

At our center, we have had experience with three types of robotic systems. First, we performed cholecystectomy with the Si and Xi systems and observed that docking was difficult. Hand movements were still uncomfortable and caused collisions of the robotic arms. Subsequently, when we attempted the newly introduced SP cholecystectomy, we found that the SP system was more convenient and ergonomic than the Si or Xi systems and continued to use this system for single-incision robotic cholecystectomy. To confirm the safety, feasibility, and convenience of the new SP system, we analyzed the experiences of all three systems at our center.

In the beginning of experiences at our center, the patient population receiving robotic surgeries primarily consisted of young female patients with a lower BMI, or a lower ASA score, who often have greater concerns in cosmesis. Since robotic cholecystectomy is a relatively new technology that requires further safety evaluation, we tended to exclude complicated cases. Due to the lack of sufficient evidence regarding the safety and effectiveness of robotic surgery, there was a possibility of increased danger to such patients. Patients who may have a higher risk of operative or postoperative complications for any surgery were typically excluded from consideration for robotic surgery. Given that the Si/Xi system was introduced before the SP system at our center, we were more careful in selecting cases during the early stages of our experience with Si/Xi. After the SP system was introduced, we believed that it was superior to the Si/Xi system in terms of improved and refined function, such as instrument articulation, and performed robotic cholecystectomy using the SP system in most cases. Thus, regarding safety, this might explain the demographic outcome that there were significantly younger patients and more females in the Si/Xi group. However, at present, there is no limitation on age or gender unless the patient disagreed with robotic surgery.

The case selection was also applied to clinically complicated cases which included patients who had undergone previous upper abdominal surgeries or preoperative ERCP or were diagnosed with severe cholecystitis. These complicated cases might increase the risk of conversion to laparoscopic or open surgery because of inflammation and adhesion. Nevertheless, there were a few cases in which robotic cholecystectomy was performed in patients with acute cholecystitis and in patients undergoing preoperative ERCP using the SP system. Almost all the procedures were successfully performed, except in one case with acute cholecystitis, which was converted to open surgery. In the study by Kang et al., significantly more cholecystectomies were also attempted in patients with acute cholecystitis using the SP than the Xi system (31.9% vs. 1.6%; *P* < 0.001)^4^, which might indicate that acute inflammation or disease severity affected the surgeons’ preference for a specific type of robotic system when performing robotic cholecystectomy. However, when deciding to perform robotic surgery, in addition to the safety of the operation, the medical costs of robotic surgery should not be ignored.

Financial and administrative issues were also factors in case selection. Robotic surgeries often cost two or three times as much as open or laparoscopic surgeries in Korea. Complicated cases have more chances of conversion surgery, which can result in additional expenses for patients without the anticipated benefits of robotic surgery. Thus, patients without personal insurance often face difficulties in accessing robotic surgeries due to financial issues. Moreover, some of them require urgent surgery, for which robotic cholecystectomy is difficult to perform due to scheduling and administrative issues. The case selection in our study aligned with those in two previous studies conducted in Korea^3,4^. It is always a concern before robotic surgery in Korea. In the future, we are planning to include more complicated cases and analyze the postoperative outcomes.

Regarding intraoperative outcomes, iatrogenic bile spillage was observed to occur more frequently in the Si/Xi group. Most of the bile spillage occurred from the gallbladder wall during the dissection of the gallbladder from the liver (cystic plate dissection). The Si/Xi system, which did not have wrist movement in its robotic arms, was limited in its ability to achieve the appropriate angle required for dissection, whereas the SP system with its multidirectional wrist movement allowed for more refined dissection, resulting in reduced bile spillage. In addition, the conversion rate in MIS is generally an important indicator of safety and feasibility. The total conversion rate was as low as 0.9%, with no significant difference between the two groups. Previous studies including patients undergoing single-incision cholecystectomy with the Si or Xi system have also reported low conversion rates of 0–3.3%^1,3,5^.

One of the most distinguishing features between Si/Xi and SP systems was the operation time. There were significant differences in all aspects of the operation time, including the docking, console, and actual dissection times. Shorter console and actual dissection times might indicate easier control of the robotic arms during cholecystectomy. Cruz et al., also reported that all three operative times were shorter with the SP system.

Because the Si and Xi systems require a skilled technique for inserting the robotic curved cannula and docking the robot arm, it could take time, and possible dislocation can result in an intracorporeal collision. In Fig. 3, the graph of the docking time in the Si/Xi system gradually decreases in consecutive cases, whereas the graph in the SP system shows a minimal change. This finding demonstrated that the learning curve of the docking in the Si/Xi system is longer compared to that in the SP system. This may be due to the structural advantage of the SP system which only requires a single arm for docking whereas the Si/Xi system requires the docking of 3 arms. In addition, the articulating function of instruments in the SP system is more ergonomic and enables easier learning and more comfortable dissection leading to a shorter dissection time and operation time. We believe that since the two systems are structurally different, previous experiences with the Si/Xi system may have had only minimal impact on the learning curve and operative times.

The postoperative outcomes, postoperative complications, and postoperative stay were comparable between the two groups. The cosmesis of the umbilical wounds in both groups was similar (data not shown). The SP system requires at least a 2.7 cm transumbilical incision to insert the robotic cannula, whereas the Si/Xi system may require an incision as small as 2.5 cm. These do not affect cosmetic results because the incision is hidden inside the umbilicus. However, the size of the incision may be associated with the rate of incisional hernia formation. In the present study, the total rate of incisional hernias was 1.5%, with no significant difference between the SP and Si/Xi groups. Previously, the rate of umbilical port site hernia after Si/Xi cholecystectomy was reported to be 5.2–8%^5–7^.

**Figure 5:**
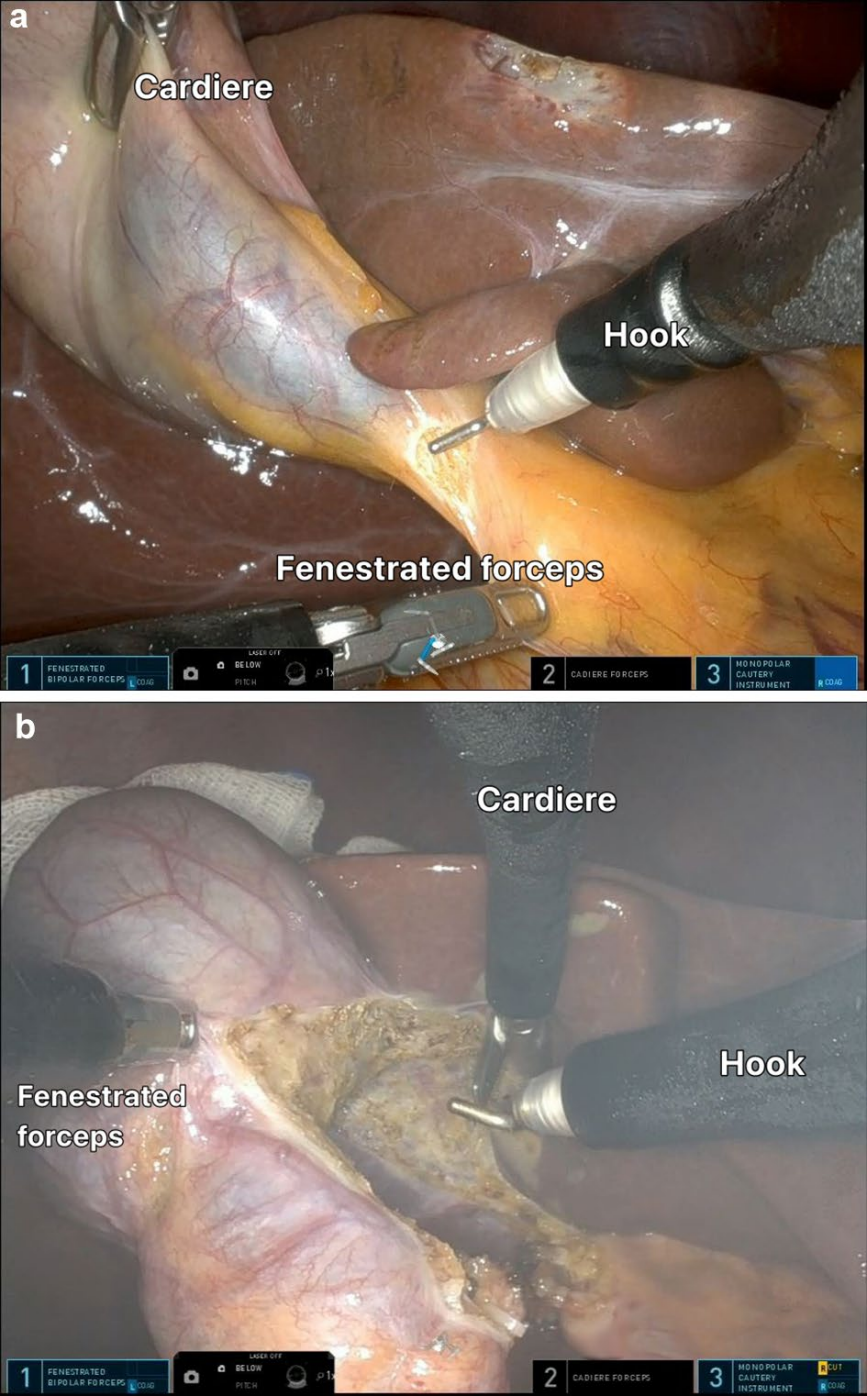
The SP system has three arms that can be controlled by the operator. The middle arm, in this case, the Cardiere forceps arm, is used for GB traction (**a**) or liver traction (**b**).

**Figure 6:**
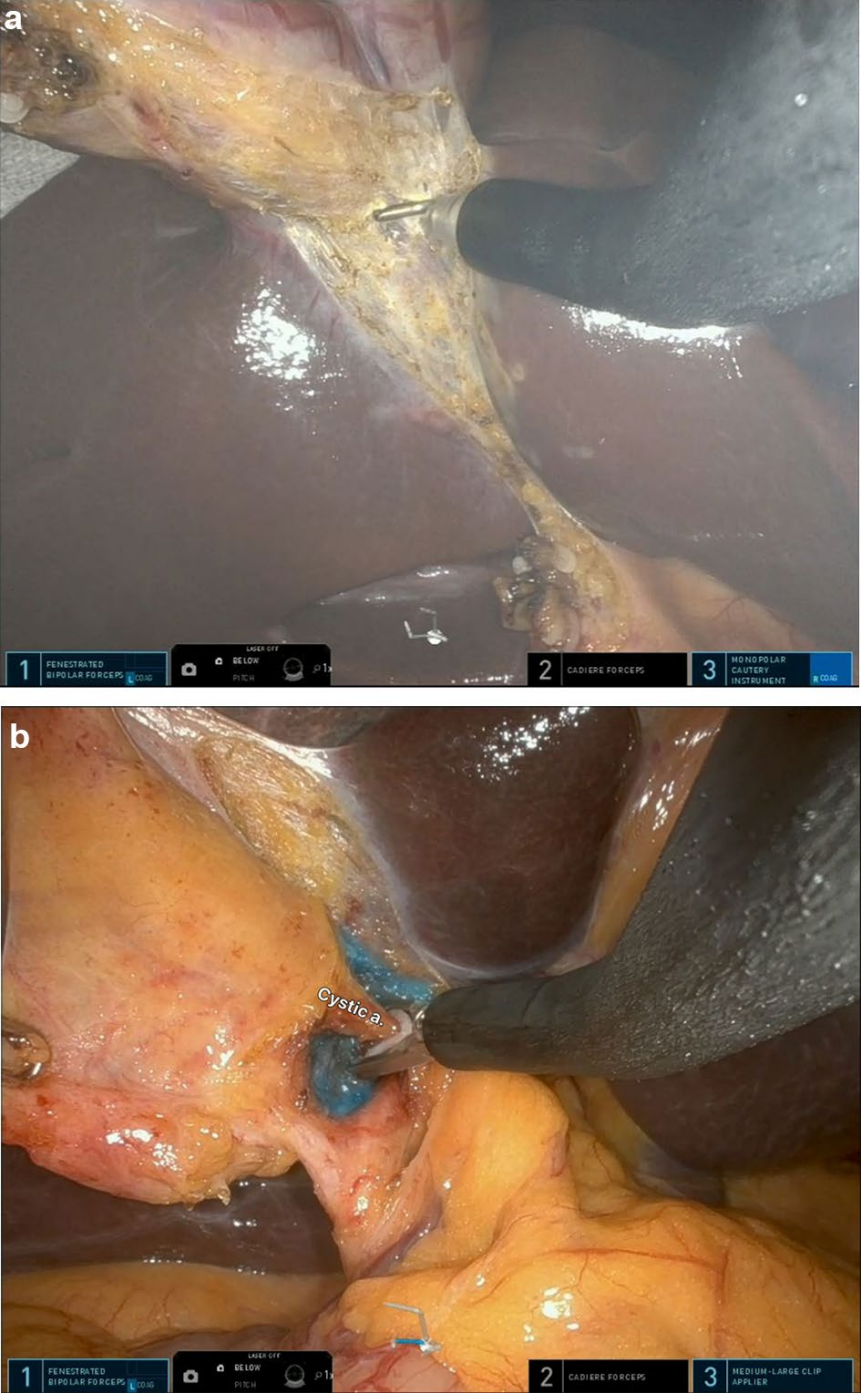
The Endo-wrist in the SP system allowed approaching the surgical field with the appropriate angle. (**a**) Hook; (**b**) hemo-lock applier.

**Figure 7:**
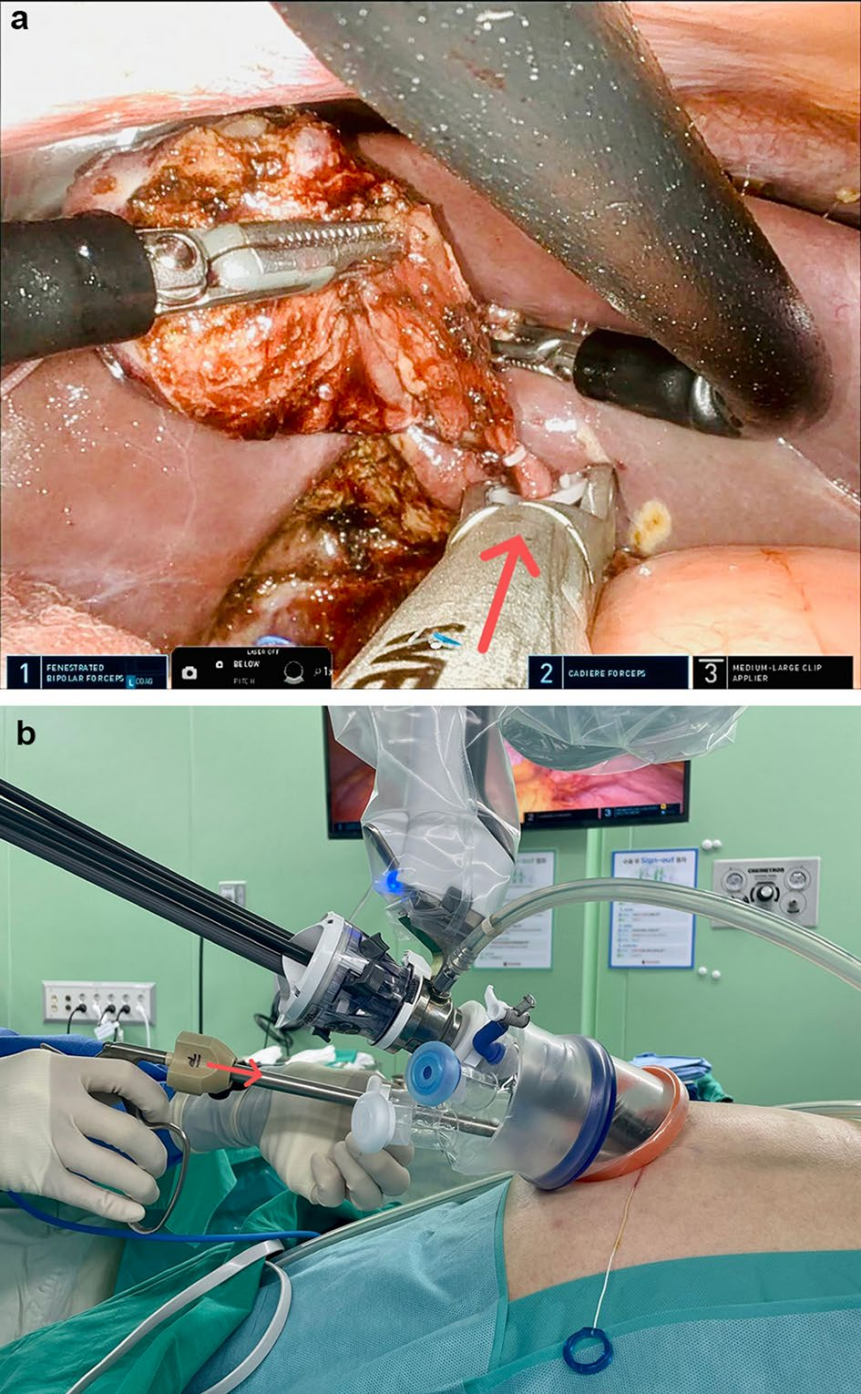
(**a**, **b**) The large hemo-lock (red arrow; “purple” size) was inserted through the umbilical port by the assistant to ligate the cystic duct.

Technically, the SP system was simpler and more convenient (Table 4). First, among the numerous advantageous features of the SP system, the third arm controlled by the surgeon for traction of the gallbladder (Fig. 5) and the multidirectional EndoWrist function (Fig. 6) are by far the most important factors for easier dissection and easier control of the GB, respectively. Second, in the case of Si/Xi, the previous study by Jung et al.^8^ introduced the reverse-port technique to perform dissection around the cystic duct and cystic artery. However, in SP, the arms can be extended using the EndoWrist. Third, in acute cholecystitis, the cystic duct can sometimes become dilated and is thus difficult to ligate using a typical single-size medium-large (green) robotic hemolock. In the SP system, the assistant can insert a larger hemolock (purple) through the gel port or other access port (ex. glove port) beside the insertion site of the SP cannula to clip the cystic duct (Fig. 7). Furthermore, if the cystic duct is too thick or accompanies Mirizzi syndrome, it cannot be ligated even with a large hemolock. However, in the SP system, because it is easier to suture with EndoWrist, primary repair can be performed on the cystic duct stump.

**Table 4:** Advantages and disadvantages of Si/Xi and SP.

	Si/Xi	SP
Advantage	3D visualization	3D visualization
	Inversion of the instruments: wider movement, better ergonomics	Easy docking process
	Curved 5 mm cannulas and semi-rigid instruments restore triangulation	Three working robotic arms Traction controlled by the surgeon
	Remote center technology minimizes collisions, crowding, and trauma	Multi-joint Endo-Wrist instruments articulation
		Distal instrument triangulation (at the tip)
		360-degree rotation
		Access narrow space
		24 cm reach
		Flipped camera view
		Broad versatile
Disadvantage	No internal wrist No triangulation at tip	No suction arms, no energy device
	Two working robotic arms Need an assistant for GB retraction	Narrow range of motion
	Difficult docking process	
	No suction arms, no energy device

However, this study has some limitations. Its retrospective nature and relatively small sample size may have limited the results. However, all robotic cholecystectomies were performed at a single center, which might help maintain the consistency of the procedure. There were case selections in both groups. Thus, either robotic systems may not be applied to certain patients who have high BMI or underlying diseases that would significantly affect the surgical outcomes.

Robotic SP cholecystectomy is safe and feasible in terms of comparable perioperative complications and low conversion rates and is convenient for many newly applied systems. We believe that robotic SP cholecystectomy is more advantageous in terms of the docking process and three robotic arms with multi-joint EndoWrist movement, which gives us reasons not to return to the Xi system.

## Conclusions

Our results and successful patient outcomes suggest that SP cholecystectomy is a safe and feasible procedure in terms of comparable postoperative complication rates and is more convenient in terms of docking and techniques. The long-term outcomes of robotic SP cholecystectomy with a larger number of cases are needed in the future.

## Data Availability

The datasets analyzed during the current study are available from the corresponding author on reasonable request.
